# Influence of the Tube Angle on the Measurement Accuracy of Peri-Implant Bone Defects in Rectangular Intraoral X-ray Imaging

**DOI:** 10.3390/jcm13020391

**Published:** 2024-01-10

**Authors:** Petra Rugani, Katharina Weingartner, Norbert Jakse

**Affiliations:** Department of Dental Medicine and Oral Health, Division of Oral Surgery and Orthodontics, Medical University of Graz, Billrothgasse 4, 8010 Graz, Austria

**Keywords:** peri-implant bone level, intra-oral radiography, implantology

## Abstract

Background: Intraoral radiography in the right-angle technique is the standard procedure to examine the peri-implant bone level in implant follow-up and implant-related studies. For the implementation of the right-angle or parallel technique, mostly ready-made image receptor holders are used. The aim of this experimental study is to analyze changes in the measurement of standardized peri-implant defects caused by a deviation in the position of the image receptor. Methods: Eleven Xive^®^ implants (Dentsply Sirona, Bensheim, Germany) were placed in bovine bone, and peri-implant defects of varying depths were created. The preparations were fixed in a specially made test stand, and intraoral radiographs were taken using the right-angle technique with standard film holders at various horizontal and vertical projection angles. Defect measurement was carried out with the imaging software Sidexis 4 V 4.3 (Dentsply Sirona, Bensheim, Germany). Results: With increasing angular deviation, larger deviations between the measured and the real extent of the defect occurred. Vertical tilting caused significant distortion, while horizontal rotation showed less effect. Conclusion: Intraoral radiography only provides a valid representation of the peri-implant bone level for follow-up or as a tool in implant-related studies if a reproducible projection direction is assured.

## 1. Introduction

Intraoral radiography using the right-angle technique is the standard procedure to examine the peri-implant bone level. It is indispensable for implant follow-up and also serves as a basis for the assessment of peri-implant bone loss and, thus, the diagnosis of peri-implantitis. It is also the preferred technique to analyze peri-implant bone level in implant-related studies [1,2]. Peri-implant inflammation is the main cause of loss of previously osseointegrated dental implants, with a prevalence of 22% (2131 patients, 8893 implants) [3]. While in peri-implant mucositis, the inflammatory cell infiltrate is limited to the supracrestal soft tissue interface, in peri-implantitis, it is spread to the bony implant site [4]. This is a serious complication that, if left untreated, will progress, leading to pain and other signs of inflammation, and may ultimately result in implant loss. Part of standardized implant aftercare is to recognize, classify, and treat early signs of peri-implant inflammation to prevent the progression to more advanced stages. Taking an intraoral radiograph to evaluate the crestal bone level is part of the strategy. It is recommended to take an intraoral radiograph using the right-angle or parallel technique at the time of installation of the prosthetic component as a baseline and to repeat this examination at regular intervals [5]. In the case of manifest peri-implantitis, the type of bone loss, i.e., whether it is an intraosseous defect or a supra-alveolar manifestation, or whether there are mixed forms [6], can already be estimated. Furthermore, the peri-implant bone level or the development and progression of peri-implantitis can be influenced by a wide variety of variables [2], including implant surface, abutment connection, prosthetic concepts, hygiene ability, etc. Consequently, radiographic imaging of the peri-implant bone is a common research method in studies in the field of implantology [7,8]. A clear depiction of the crestal bone level and the verification of the existence and/or progression of bone loss in periodically repeated radiographs with the same projection geometry are essential for the comparison to earlier images or other implants. Particularly in scientific studies, this is of utmost importance [9,10,11].

In daily dental practice, intraoral radiographs represent the standard tool to assess the crestal bone level around dental implants. The spatial relationship between the beam source, object, and image receiver essentially determines the two-dimensional radiograph formation during the recording. The projection of three-dimensional defects onto two-dimensional image receptors is associated with typical misrepresentations. These include:

- Image enlargement through projection magnification;
- tilting of the examination objects, misinterpretation of the edges of the examination object/sites on the image; and
- an inaccurate evaluation method for distance measurements within the image [12].

In order to obtain representations that are as accurate as possible, it is recommended to reduce the influence of these factors as much as possible. Images are taken using standardized film holders in combination with a right-angle or parallel technique [13]. The image receptor is aligned via the film holder at a defined angle to an indicator ring, onto which the tube is placed flush. This means that the central beam is always perpendicular to the image receptor axis. In addition, the holder should be aligned with the patient’s bite on a bite plate so that the image receptor is positioned parallel to the tooth or implant axis [1]. Even if this is not often the case, at least a reproducible alignment of the central beam with the object axis should be achieved for the correct assessment of changes in the crestal bone level. Since reference values such as the implant length are often known, measurements can also be made, and thus a quantitative analysis of changes over time can be carried out. These measurements usually take place in the submillimeter range [14]. A weakness of this method is the uncertainty of how the film holder is placed and deflected by the patient’s bite, which is not a constant parameter and can be different with each examination. Being able to estimate the dimensions of such deviations is important for assessing the accuracy of the image. Since calculating geometric distortions cannot be established in everyday life, guideline values are of great importance [1].

The aim of this experimental study is to assess the effect of deflection of the right-angle holder on the measurement results of standardized peri-implant defects.

## 2. Materials and Methods

**Figure 1:**
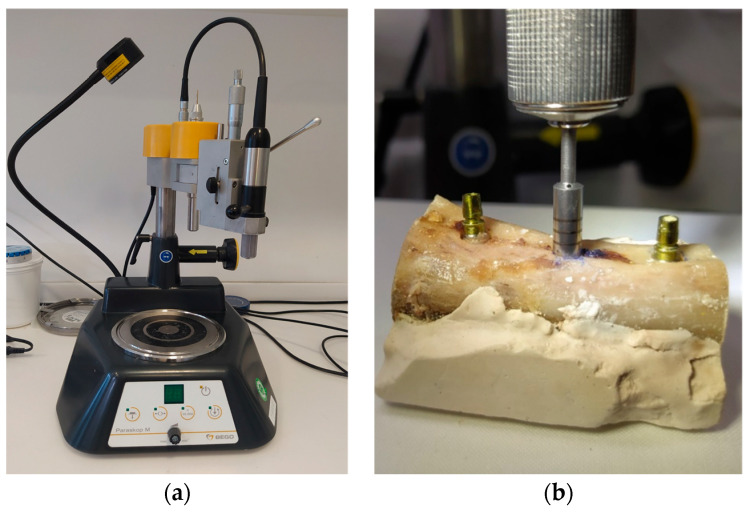
Implant placement and defect creation (**a**) Drill press to maintain the exact drilling direction. (**b**) Creation of defect around a previously inserted implant with the trephine bur.

Uncooked bovine ribs were used for the implantation because they have the same properties as a human jawbone. The ribs were encased in SnowWhite plaster to ensure stability. As a next step, eleven Xive^®^ implants (Dentsply Sirona, Bensheim, Germany) with a diameter of 3.8 mm and a length of 11 mm were placed in the bones. The implantation was carried out according to the protocol specified by the manufacturer. All drilling was carried out with a fixed drill bit, which ensured that the identical axis could be maintained exactly every time. (Figure 1a) Subsequently, implants were placed. Insertion torque exceeded 25 Ncm for all implants.

After implant insertion, a circumferential defect was created using a trephine bur in the implant axis. Eleven defects with depths of 2, 2.5, 3, 4, 5, 6, 7, 8, 9, 10, and 11 mm were created. (Figure 1b) Water cooling was applied throughout the complete drilling process.

**Figure 2:**
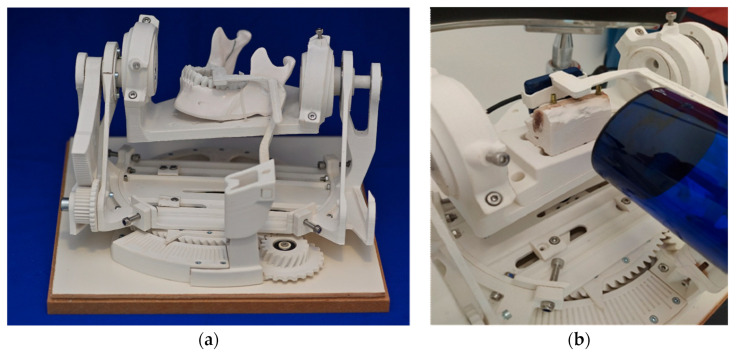
Experimental setting (**a**) test stand. (**b**) Prepared bovine ribs and positioned radiographic film rigidly fixated to the radiation tube.

To ensure accurate and repeatable measurements with exactly defined parameters, a test environment was created (Figure 2a).

The mechanics contained here made it possible to change the angle between the radiation tube and the test objects in precise, predefined steps. By this, a biangular deviation in the vertical and horizontal directions was imitated. (Figure 2b) The displacement of the angle was minus 15 degrees to plus 15 degrees in steps of 5 degrees in the vertical plane and minus 8 degrees to plus 8 degrees in steps of 3 degrees in the horizontal plane. The test stand was designed by a technical engineer (Konrad Felix Fellner e.U., Graz, Austria) in the programs Rhino V4^®^ (Robert McNeel & Associates, Seattle, Washington, DC, USA) and Solidworks 2017^®^ (Dassault Systèmes SolidWorks Corporation, Waltham, MA, USA).

The individual parts were manufactured through 3D printing using the Fused Filament Fabrication additive process. Polyactic acid 3D printing filament (PLA) was chosen as the material because of its good physical properties and easy availability. PLA is largely resistant to most common solvents and does not affect X-rays. The used cylinder head screws and ball bearings were kept outside the region of interest. The characteristics of the test stand were:

- A platform for fixing the objects to be X-rayed
- Adjustment options for adjusting the object to the respective axis pivot points
- Two independently rotatable axes
- Angle markings for referencing, measurement, and data collection
- An adjustable holder for the X-ray sensor and X-ray machine

The platform, to which the test object was fixated, was positioned at an angle in the room and held by appropriate components so that it could be moved freely. This changed the projection angle of the rigidly positioned central beam.

**Figure 3:**
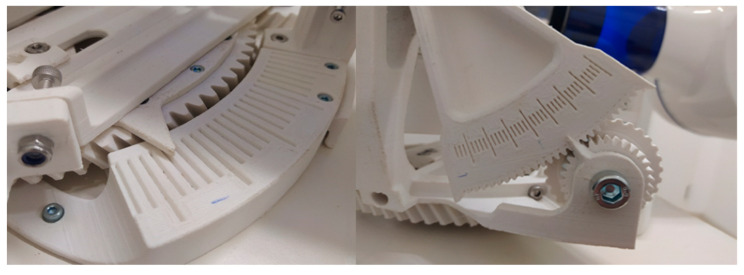
Mechanism to adjust horizontal and vertical angulation.

The horizontal rotation of the platform allowed seven different settings with the angular positions −8°, −5°, −3°, 0°, 3°, 5°, and 8°. The vertical axis allowed the angle settings of −15°, −10°, −5°, 0°, 5°, 10°, and 15°. (Figure 3) The ribs encased in plaster were positioned centrally on the platform to allow free movement in all directions.

The X-ray tube (Heliodent Plus, Dentsply Sirona, Bensheim, Germany) was set in a zero-degree position parallel to the holding arm of the digital sensor at a distance of 8 cm from the image receptor. The fixed models were imaged radiographically with a tube current of 7 mA, a tube voltage of 60 kV, and an exposure time of 0.06 s using the right-angle technique.

The Sidexis 4 V 4.3 imaging software (Dentsply Sirona, Charlotte, NC, USA) was used to measure the depiction of the defects on the radiographs. The measurement tool was calibrated based on the implant length of 11 mm.

Every defect was traced individually using the Sidexis measurement tool parallel to the implant axis, starting from the crestal bone edge. Defects were 2 to 11 mm deep. Each defect was pictured 49 times in different directions, ranging from −15 to +15 degrees in the vertical dimension and from −8 to +8 degrees in the horizontal dimension.

The data were collected in an Excel^®^ spreadsheet. (Microsoft 365, Version 2207 (Build 15427.20194), Microsoft, Redmond, Washington, DC, USA). The deviation of the measuring depth from the true depth of the defect was calculated. Statistical analysis to assess the results included ANOVA and regression analysis using SPSS software (IBM SPSS Statistics 26.0, IBM Corporation, Armonk, NY, USA) at a 5% significance level.

## 3. Results

**Figure 4:**
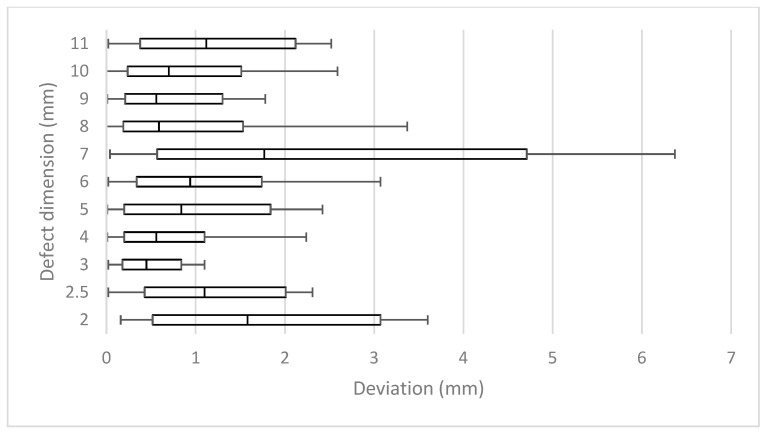
Mean measurement errors of all 11 defects (2.5–11 mm) of all horizontal and vertical deflections.

**Figure 5:**
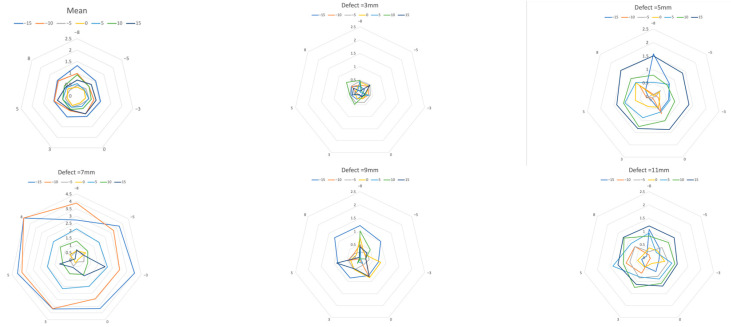
Measurement error due to vertical deviation of the projection. Mean over all defects and defects with 3, 5, 7, 9, and 11 mm. A greater vertical deviation (−15 to 15 degrees, colored lines) of the projection direction led to a larger measurement error. (Each mesh section equals 0.5 mm, with horizontal rotation at the corner points of the mesh.) Note the extreme outliers in the 7 mm defect.

Altogether, 539 images were analyzed. The deviation of the measured dimensions of the depicted defects from the known true defect depths was 0.0 to 4.6 mm (mean 0.7 ± 0.7 mm) (Figure 4 and Figure 5).

When viewed in terms of the length of each individual defect, the mean results of each defect differed in relation to the correspondent real depth between 7% and 28%, respectively, from 0.34 mm to 1.34 mm (mean 0.70 ± 0.24). This resulted in mean deviations of 15.2% (± 5.0%).

Vertical tilting alone resulted in mean deviations of 0.34 to 1 mm (mean 0.65 ±0.26 mm) or 7% to 20% (mean 13.9 ± 5.4%) (Table 1). In the horizontal plane, measurements differed from real lengths by 0.34 mm to 0.5 mm (mean 0.42 ± 0.06) or 8% to 10% (mean 8.6 ± 1%) (Table 1).

The deeper the defect, the greater the deviation that appeared when the angle changed horizontally and vertically. The smallest deviation from the actual length in the range of seven to 10% was found in the vertical zero-degree position, even with horizontal angular rotation. The more the vertical angle differed from the zero-degree position, the greater the mean deviation. (Table 1, Figure 5) On the other hand, differences barely increased in the horizontal direction, as the rotation increased.

An ANOVA analysis of variance showed that the influence of the vertical tilt was highly significant, but the results for the horizontal twist were not. Furthermore, Pearson’s correlation coefficient showed a positive correlation between vertical tilt and measurement error. An exception were the results of the 7 mm defect, some of which showed noticeably high outliers (Table 2).

A regression analysis also confirmed a significant connection between vertical tilting and the resulting measurement difference (*p* < 0.001), but none for horizontal rotation (*p* = 0.372).

**Figure 6:**
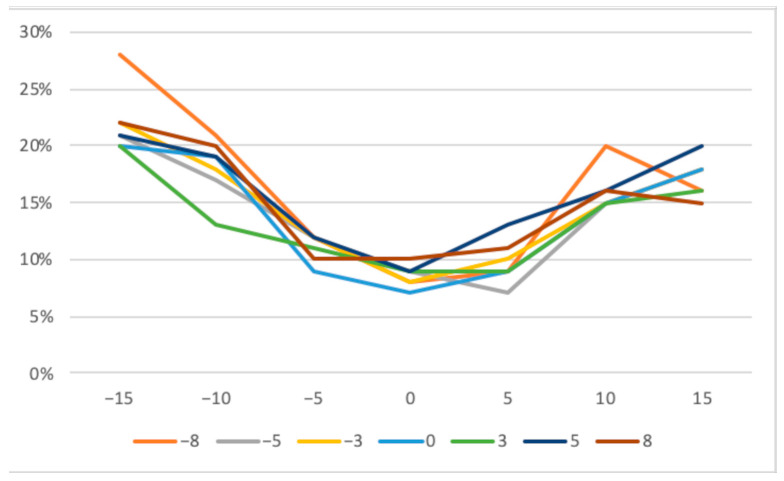
Mean measurement deviations across all defects with horizontal (−8 to +8 degrees) and vertical tilting (−15 to +15 degrees).

The largest average deviation of the defects occurred at the −8° horizontal and −15° vertical degree settings. Rotating in the negative degree range brought the defect closer to the radiation source. At the same time, the distance to the image sensor increased. Consequently, distortion was more pronounced and measured lengths, and therefore, the differences to the real lengths were larger in all values of the negative direction of rotation (Figure 6).

**Figure 7:**
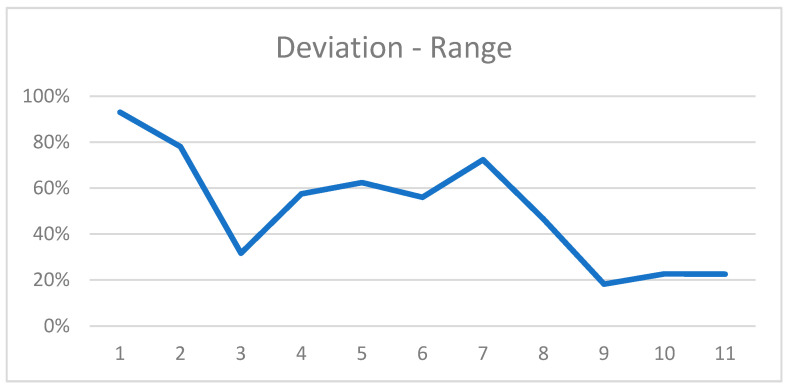
Range of most extreme measurements for all defects in relation to the defect dimension.

The mean range from the shortest to the longest measurement of a defect was 2.61 mm (±1.12 [0.95; 5.06]) or 51% (±25% [18.2%; 93%]) in relation to the depth of the corresponding defects (Figure 7).

## 4. Discussion

The study demonstrated that tilting of prefabricated right-angle holders led to measurement errors when evaluating peri-implant bone defects in intraoral radiographs. A vertical deflection resulted in a greater length deviation than a horizontal deflection. Although horizontal rotation alone had no real effect on the measurement deviations, an even greater deviation was evident when horizontal rotation was correlated with vertical rotation. The results deviated from the true defect size by an average of 15.2%. The maximum range of deviations occurred in the 7 mm defect, where the shortest and longest measurements differed by 5 mm; the mean range was 2.61 mm (±1.12 [0.95; 5.06]) or 51% (±25% [18.2%; 93%]) of the defect size. Nevertheless, considering the wide distrubtion of measurements with extreme outliers in the 7 mm defect, errors in execution must be considered probable. To the best of our knowledge, there is only one comparable experimental study that calculated a 11.4% distortion if the film holder is tilted 30% in the vertical direction [10,15].

There are several imaging modalities that can be used for radiological diagnostics in dental practice. Since the turn of the millennium, the classic procedures of intraoral radiography and panoramic radiography have been joined by cone-beam-computed tomography (CBCT), which allows in-office cross-sectional diagnostics. Before CBCT was established, this was mainly carried out using computed tomography, for which patients had to be referred to a radiology institute or to a specialized radiologist. The advantages of cross-sectional imaging are apparent. While in two-dimensional imaging, the structures that lie one behind the other in the beam path are superimposed, cross-sectional imaging makes it possible to display the structures separately from one another. In the case of peri-implant defects, this means that the buccal and lingual parts of the peri-implant bone, which can also be affected by an osseous lesion, can potentially also be visualized.

In the case of peri-implant bony defects, this means the three-dimensional extent of the defect and the parts that are located buccaly or orally to the implant, as well as revealing fenestration and dehiscence defects, might be better depicted [16,17]. Studies by Ritter et al. and Steiger-Ronay et al. concluded that measurements of distal and mesial bone levels are equally accurate with intraoral radiography and CBCT [18,19].

Kühl et al. compared the diagnostic capacity of different dental imaging modalities in the depiction of peri-implant defects in a cadaver study [20]. Six implants were inserted into a human edentulous lower jaw. Defects with known depth and three-dimensional configuration were created. Conventional intraoral radiography in parallel technique using a film holder, digital panoramic radiography, cone-beam-computed tomography with a volume of 8 × 8 and a voxel size of 0.125, and computed tomography with a slice thickness of 0.72 mm were employed. For intraoral radiography, CBCT, and computed tomography, the mandible was fixated in an individualized Plexiglas panel.

In their study, intraoral radiography performed best, with the highest sensitivity and specificity over all defects, even though only the mesial and distal bone surrounding the implant could be depicted. Furthermore, despite superimposition and distortion, panoramic radiography yielded the best results in determining the defect type (two-, three-, or four-wall defect). This was even the case in two-wall defects, where the buccal and oral parts of an implant—areas that are usually not clearly shown on two-dimensional images—are affected (classification of Renvert and Giovannoli 2012 [21]).

On the other hand, panoramic radiography showed the worst sensitivity for 1 mm defects. These small defects were discovered best by CBCT, but CBCT also showed the lowest specificity for 3 mm defects. Computed tomography yielded the lowest overall sensitivity.

Consequently, despite the technology-specific shortcomings of two-dimensional imaging, conventional or digital intraoral radiography is still the gold standard for picturing the peri-implant bone [2,22]. It is a widely accepted technique for the long-term evaluation of marginal bone changes, especially at interproximal sites, of osseointegrated implants [23]. For this, there are several main reasons: First, the high sensitivity and specificity in depicting osseous lesions of all dimensions, as mentioned above, are the most important capacities of intraoral radiography. This is probably due to the high spatial resolution of the technology, with 10 to approximately 25 line pairs per mm (lp/mm), whereas three-dimensional imaging techniques display only 1–2 lp/mm or even less (computed tomography) [24]. Further, the widespread availability and cost-effectiveness of intraoral radiography are supporting its practicability and, consequently, its daily use. The possibility of standardized image acquisition through the use of image holders allows use in the area of years of postoperative follow-up. And last but not least, intraoral radiography is associated with the lowest patient exposure to radiation of all dental imaging modalities [25,26]. In general, the long-cone paralleling technique, supported by positioning devices, is used. For posterior intraoral radiographs with the widely used photo-stimulable phosphor storage, a radiation dose of 1.2 µSv to 2 µSv arises [27].

Despite the low patient doses in dental radiography, the International Commission on Radiological Protection (ICRP) considers the linear non-threshold model when recommending radiation safety guidelines. The cumulative effects of low-dose radiation could trigger cytotoxic alterations and genetic instability in sensitive tissues and organs [28]. Dentoalveolar cone-beam-computed tomography (CBCT) has been advocated as a superior radiologic method but is potentially linked to higher radiation doses of 11–674 µSv [25,29]. The three-dimensional depiction of defects might offer additional information. This could be particularly beneficial in the detection of small defects or the planning of invasive procedures. Nevertheless, it has to be considered that titanium implants cause extensive metal artifacts. The depiction of the implant is increased in size by 12–15%, and consequently, the buccal bone is clearly visible and reduced by about 0.3 mm, which makes the dentist’s assessment with respect to implant-bone density and thickness massively difficult [30,31]. This is probably the main difference in imaging defects around implants compared to defects around natural teeth. Therefore, CBCT should not be used routinely to assess peri-implant bone defects [32].

In summary, two-dimensional imaging (e.g., periapical or panoramic radiography in cases with multiple implants) should be the first selection criterion for the assessment and monitoring of bone levels around the implant following its placement and osseointegration [33].

Many factors can influence the peri-implant bone level. The most common cause of crestal bone loss is peri-implantitis. The definition of this infection-related inflammatory disease was not uniform until the World Workshop on the Classification of Periodontal and Peri-implant Diseases and Conditions, which took place in Chicago (USA) in November 2017. The World Workshop was co-sponsored by the American Academy of Periodontology (AAP) and the European Federation of Periodontology (EFP) [34].

Since then, peri-implantitis has been defined by the following triad:

- Bleeding and/or suppuration on gentle probing;
- Increased probing depth compared to previous examinations;
- Bone loss.

If no baseline information is available, the following findings may indicate peri-implantitis:

- Bleeding and/or suppuration on gentle probing;
- Probing depths of ≥6 mm;
- Bone level ≥ 3 mm apical to the most coronal section of the intraosseous implant portion.

But even before this classification, all definitions included, in varying degrees, the loss of the alveolar jawbone. In the case of manifest peri-implantitis, the intraoral radiograph is used, among other things, not only to diagnose the disease but also to assess the further course or the success of the therapy. It is recommended to perform a baseline radiograph at the timepoint of installation of the prosthetic component as an initial reference to which further images are compared in follow-up [35].

Other possible factors influencing the peri-implant crestal bone level are mechanical stress, especially in the case of occlusal miscontact or bruxism, or inflammatory stimuli due to the materials used or the nature of the implant-abutment connection [36,37,38].

The therapeutic protocol, such as the time of implantation, the healing time, and the healing conditions, may also have an impact [14,36]. These factors are scientifically investigated in controlled clinical studies. In these studies, the intraoral right-angle radiograph is often used as a diagnostic method, and ready-made image holders might have been used. The results are mostly in the millimeter, not infrequently even in the submillimeter range [8,39].

A recent study demonstrated high inter-observer consistency in bitewing images of high quality [40]. The high spatial resolution and low radiation dose are ideal for the necessary repeated recordings over many months and years. Furthermore, the quality of cross-sectional imaging is substantially limited around dental implants due to metal-related extinction or beam-hardening artefacts. For this reason and also due to lower spatial resolution and higher radiation dose, cross-sectional cone-beam-computed tomography is not routinely used to examine the peri-implant bone. On the other hand, the superiority of CBCT in the assessment of peri-implant bone defects was demonstrated in a dog study [41]. Especially vestibular dehiscences could not be visualized on the intraoral radiographs.

Intraoral radiographs are summation images with the typical associated shortcomings: The superimposition of all objects between the radiation source and the image receptor, since the beam of rays spreads out in a cone shape, the magnified depiction of the imaged object, and the image distortion, or unequal magnification in spots, owing to the oblique position of the object. The extent of magnification is directly connected with the position of the image receptor and its distance to the radiation source, or with the ratio of the film-focus distance to the focus-object distance. This aspect was not particularly addressed in the experimental setting of the presented study because the film focus distance was a fixed value. In the oral cavity, the possible causes of a tilted object or film position as a cause of distortion are varied. In the maxilla, a strong inclination of the palate and/or a low palate, and in the mandible, interference with the floor of the mouth may force the image receptor out of its ideal position [1].

Furthermore, uncertainties in the determination of the crestal reference point for length measurements might cause measurement errors. The depiction of the crestal level of the peri-implant bone could be ambiguous due to the superimposition of bone images corresponding to the crest on the buccal and the lingual sides that may not coincide.

Consequently, the correct recognition of the reference points at the interface between the alveolar bone and the implant might be difficult [1].

In order to at least minimize the importance of these influencing factors, identical recording conditions are crucial to ensuring that the images are comparable. Commercially available image receptor holders are intended to ensure these reproducible conditions. The radiographic film or sensor is rigidly fixed in these holders, thereby achieving a constant right angle to the central beam of the radiation source if the X-ray tube is positioned correctly on the holder. In addition, the holder has a bite block that is used to position it in relation to the depicted object. When the patient bites down on the block, the sensor should align itself parallel to this object, usually a tooth or an implant.

The key problem is that the final bite position of each individual patient is not exactly determined by the design of such holders. Deviations in the contact surface or twisting or tilting of the holder are easily possible in the daily routine and may be different each time. For example, anatomic obstacles such as the tongue or the hard palate may prevent the holder from aligning itself perpendicular to the object. This intended position is also not reached if the patient does not close the mouth completely, for example, due to pain or gagging. If the contact area is small and the position of the bite block is therefore unstable, the risk of tilting is even greater.

About 20–30 years ago, the distortion of intraoral radiographs showing periodontal defects was a common topic in scientific papers [42,43,44,45,46,47,48]. Back then, in vivo comparison with measurements of defects during periodontal surgeries was the gold standard to examine the accuracy of measurements on intraoral radiographs [43]. The horizontal and vertical angulation differences of the central beam from the orthoradial projection and radiographic magnification were calculated, for example, by interpreting the depiction of the wires placed in known distances from the film holder [49,50]. It has to be considered that many factors may cause biases when interpreting in vivo measurements. Defects are irregularly shaped, and reference structures are not always clearly shown. The deflection of the central beam is not known but calculated. These factors add up to deviations that can additionally occur when interpreting the images.

The experimental setting of the presented study, with uniform defects of known length and fixed deflections of the central beam, ruled out some of these uncontrollable influences.

The results demonstrated that deviations in the tenth of a millimeter range can easily occur if the image holder is twisted or tilted, even under these idealized conditions. The limitations of this study are due to its experimental setting. Measurement errors might even be greater in real-life situations, where additional factors like the local anatomy might exert an additional influence. These factors might lead to inconsistencies in the technical parameters of the image acquisition, like the film-focus distance or even the more pronounced tilting of the image holder. Real-life peri-implant defects come in various configurations, and the identification of a reference point might be difficult [49]. Furthermore, deviations in the interpretation of the radiographs might occur. These aspects were not addressed in this study, which means that the magnitude of the measurement errors could be underestimated.

Therefore, results obtained with the use of pre-fabricated right-angle image holders to assess the peri-implant bone level have to be interpreted with caution. The lack of reproducibility of the measurement conditions is even more relevant than the deviation of the measurement compared to the actual defect size. As part of studies, the production of individually adapted image holders might assure identical alignment of the image receptor at each radiographic examination [51,52,53,54]. The bite block is individually adjusted using silicone or plastic materials.

However, there are a few points to consider when making such holders with individualized bite blocks.

Soft and flexible materials, such as silicone, may be more susceptible to incorrect positioning, especially if they are only supported on one side. These inaccuracies can mean that a better representation of the crestal bone level cannot be achieved with such holders [1].

Designing bite blocks with a larger support or that cross the dental arch might help increase the accuracy of the method. Sadek et al. demonstrated in a study comparing two images taken at three-month intervals in vivo that measurements using individual X-ray positioning stents provided more precise and reproducible images than those fabricated with conventional film holders [55].

## 5. Conclusions

The assessment of the peri-implant bone level with intraoral radiographs may be biased if ready-made image holders are used. Vertical tilting of the image holder may result in distortion of images in the tenths of a millimeter range. The use of individualized film holders may produce comparable results over a longer period of time and thus ensure comparability between implants in scientific studies on the influence of various biological or technical parameters on bone healing or the peri-implant bone level.

## Figures and Tables

**Table 1:** Relative mean deviations in regard to deflection across all 11 defects. (columns—horizontal deflection (−8° to +8°), lines—vertical deflection (−15° to +15°)).

Deflection	−8	−5	−3	0	3	5	8
−15	28%	21%	22%	20%	20%	21%	22%
−10	21%	17%	18%	19%	13%	19%	25%
−5	12%	12%	12%	9%	11%	12%	10%
0	8%	9%	8%	7%	9%	9%	10%
5	9%	7%	10%	9%	9%	13%	11%
10	2%	15%	15%	15%	15%	16%	16%
15	16%	18%	18%	18%	16%	20%	15%

**Table 2:** Mostly positive correlation of vertical tilting to measurement deviation.

Defect	2 mm	2.5 mm	3 mm	4 mm	5 mm	6 mm	7 mm	8 mm	9 mm	10 mm	11 mm
PCC *	r = 0.177	r = 0.857	r = 0.190	r = 0.619	r = 0.734	r = 0.607	r = −0.606	r = 0.803	r = 0.478	r = 0.094	r = 0.827
	*p* = 0.224	*p* < 0.001	*p* = 0.192	*p* < 0.001	*p* < 0.001	*p* < 0.001	*p* < 0.001	*p* < 0.001	*p* = 0.519	*p* = 0.519	*p* < 0.001

* Pearson’s correlation coefficient (PCC).

## Data Availability

The data that support the findings of this study are available from the corresponding author upon reasonable request.
